# Nipocalimab in Early‐Onset Severe Hemolytic Disease of the Fetus and Newborn

**DOI:** 10.1002/pdi3.70067

**Published:** 2026-09-24

**Authors:** Aditya Hemedra Bhatt, Somashekhar Marutirao Nimbalkar, Dipen Vasudev Patel, Reshma Kushal Pujara

**Affiliations:** ^1^ Department of Neonatology Pramukhswami Medical College Bhaikaka University Karamsad Gujarat India; ^2^ Department of Pediatrics All India Institute of Medical Sciences Deoghar Deoghar Jharkhand India

## 1 Introduction

Severe hemolytic disease of the fetus and newborn (HDFN) is a potentially life‐threatening condition that occurs when maternal alloantibodies target fetal erythrocytes, leading to fetal anemia [1]. In severe cases, this condition can result in hydrops fetalis (fluid accumulation in fetal tissues), stillbirth, or severe preterm birth. Early‐onset HDFN, defined as a history of severe HDFN requiring intrauterine transfusion (IUT) or causing fetal loss at ≤ 24 weeks of gestation, represents a particularly high‐risk phenotype associated with significant mortality and morbidity. This strict definition, consistent with the inclusion criteria of the Phase 2 UNITY study (NCT03842189) trial, highlights a population often excluded from standard management protocols due to the technical challenges of extremely preterm IUTs [1, 2].

Nipocalimab targets the neonatal Fc receptor (FcRn), which plays a dual role in HDFN pathology: it recycles IgG within maternal endothelial cells, protecting IgG from lysosomal degradation, and mediates the active transport of IgG across the placenta to the fetus. By blocking the IgG‐binding site on FcRn, nipocalimab accelerates the catabolism of maternal IgG antibodies, including pathogenic alloantibodies, while simultaneously inhibiting their transplacental transfer into the fetal circulation. This dual mechanism effectively reduces the fetal burden of maternal antibodies more potently than therapies that rely solely on dilution or saturation effects. This article summarizes and discusses the findings of a previously published Phase 2 trial that investigated the efficacy and safety of nipocalimab in pregnancies at high risk for recurrent early‐onset severe HDFN [3].

### 1.1 Study Design and Methods

The study was designed as an international, open‐label, and single‐group, Phase 2 clinical trial, published in the New England Journal of Medicine in August 2024 [3]. The participants were pregnant women who had previously experienced an early‐onset severe HDFN event, typically defined by fetal anemia, fetal hydrops, or stillbirth linked to alloimmunization. A total of 13 participants with a high risk of recurrent HDFN were enrolled across 8 centers in seven countries [3] (Figure 1).

**FIGURE 1:**
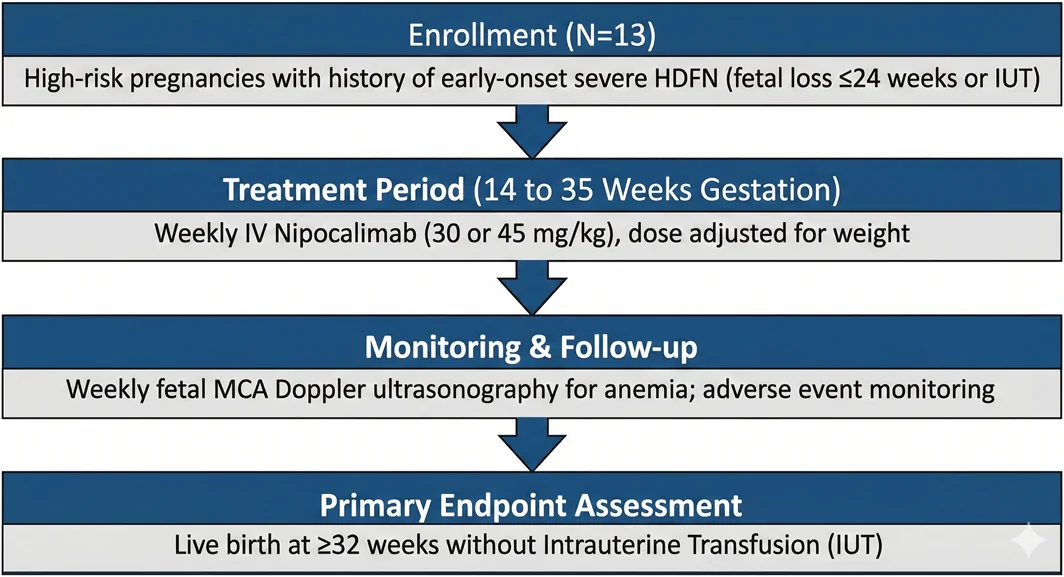
Study design of the Phase 2 UNITY trial. Thirteen pregnant participants at high risk of recurrent early‐onset severe hemolytic disease of the fetus and newborn (HDFN), based on a previous fetal loss at or before 24 weeks of gestation or a prior intrauterine transfusion (IUT), received weekly intravenous (IV) nipocalimab (30 or 45 mg/kg, adjusted for maternal body weight) from 14 to 35 weeks of gestation. Participants underwent weekly fetal middle cerebral artery (MCA) Doppler ultrasonography and adverse‐event monitoring. The primary endpoint was live birth at 32 weeks of gestation or later without IUT. Schematic created by the authors based on the UNITY trial [3].

The treatment regimen involved administering intravenous nipocalimab at a dose of either 30 mg/kg or 45 mg/kg of maternal body weight weekly from 14 to 35 weeks of gestation. Dose adjustments were made during the trial in response to weight changes during pregnancy, ensuring consistent FcRn blockade coverage throughout the treatment period. Participants were monitored for adverse events, and fetal anemia was closely tracked using Doppler ultrasonography of the middle cerebral artery (MCA), which serves as a biomarker for anemia in the fetus. Importantly, fetal surveillance followed a standardized protocol: participants underwent weekly MCA Doppler assessment, and intrauterine transfusion was performed only after cordocentesis‐confirmed fetal anemia, defined by an MCA peak systolic velocity of at least 1.5 times of the median. The treatment period aimed to extend to 35 weeks of gestation, after which delivery was planned or induced if needed.

The study’s primary endpoint was the live birth of a fetus at 32 weeks of gestation or later without the need for intrauterine transfusions. Secondary endpoints included gestational age at delivery, the number of intrauterine transfusions required, the occurrence of fetal hydrops, and neonatal outcomes (including the need for exchange transfusions or simple erythrocyte transfusions).

## 2 Results

The UNITY trial demonstrated that nipocalimab significantly improved the outcomes for pregnancies affected by early‐onset severe HDFN.

Given the rarity of early‐onset severe HDFN and the ethical implications of withholding standard care, a concurrent placebo control was not feasible. Instead, the study utilized a pre‐specified historical benchmark of 10% for treatment success. This benchmark was derived from extensive historical data indicating that in pregnancies with a history of severe HDFN (loss or IUT < 24 weeks), the rate of live birth without IUT is effectively 0%.

The historical benchmark was 0%, with a prespecified clinically meaningful difference of 10%. In the UNITY trial, 7 of 13 participants (54%) achieved the primary endpoint, which was significantly higher than the prespecified 10% clinically meaningful threshold (*p* < 0.001). This statistical significance validates the therapeutic efficacy of nipocalimab in this high‐risk population, demonstrating a capability to prevent severe fetal anemia that far exceeds the outcomes expected under the current standard of care (Figure 2).

**FIGURE 2:**
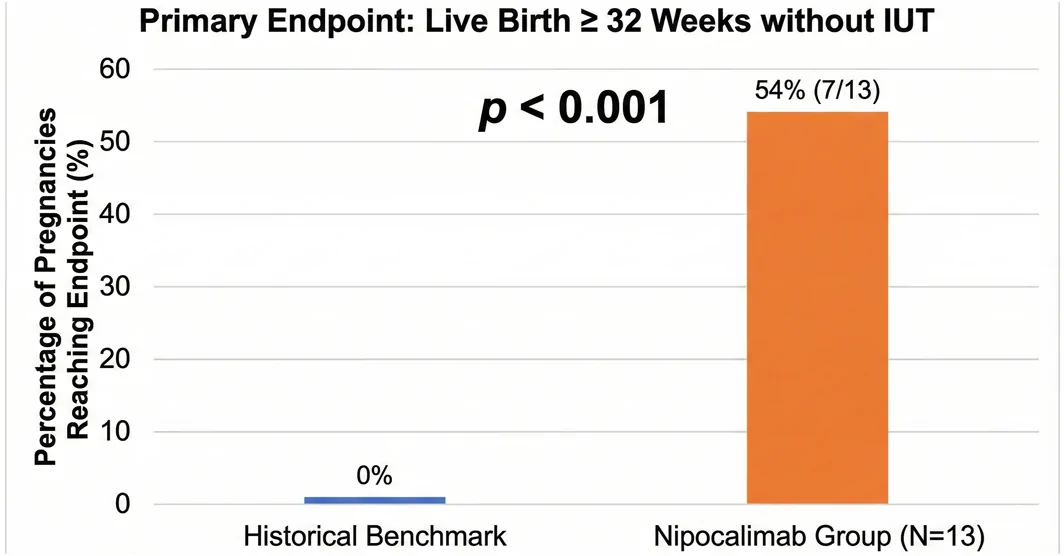
Primary efficacy endpoint in the Phase 2 UNITY study. Seven of 13 nipocalimab‐treated pregnancies (54%) resulted in live birth at 32 weeks of gestation or later without intrauterine transfusion (IUT), compared with 0% in the participants’ qualifying historical pregnancies. The observed response also exceeded the prespecified 10% success benchmark (*p* < 0.001). IUT, intrauterine transfusion. Data derived from Moise et al. [3].

Secondary analyses revealed substantial improvements in disease severity and neonatal morbidity compared to the participants’ obstetric histories.

- Elimination of Hydrops Fetalis: In the participants’ qualifying historical pregnancies, hydrops fetalis—a condition associated with high perinatal mortality—was present in 54% (7/13) of cases. In contrast, no cases of hydrops fetalis (0/13) were observed in the pregnancies treated with nipocalimab.
- Delay of Intervention: Among the 6 participants who eventually required IUT, the median gestational age at the first procedure was 27 weeks and 1 day. This represents a clinically critical delay of approximately 5–7 weeks compared to the historical median of 20–22 weeks observed in similar cohorts. This delay bridges the fetus past the gestational window of < 20 weeks, where IUT procedures carry a significantly higher risk of fetal loss (approximately 17%–20% per fetus) compared to later gestation (< 2%).
- Reduction in Transfusion Burden: Nearly half of the neonates (46%; 6/13) required no transfusions (intrauterine or postnatal) of any kind. Furthermore, among the 12 live births, only one infant (8%) required an exchange transfusion, a procedure frequently necessitated in severe HDFN management to prevent bilirubin encephalopathy.
- Pharmacodynamic Confirmation: The clinical benefits correlated with the drug’s mechanism of action—maternal IgG levels were reduced by approximately 85% by 18 weeks and remained suppressed, whereas neonatal IgG levels at birth were roughly 60% of maternal levels, confirming effective blockade of transplacental antibody transfer.

### 2.1 Safety Profile

Although the authors describe the safety profile as manageable, the burden of adverse events warrants careful consideration. Serious adverse events were reported in 38% of mothers; most were considered consistent with the underlying HDFN, pregnancy‐related complications, or prematurity rather than being directly attributable to nipocalimab. Although the majority of these events were attributed to the underlying obstetric complications of HDFN rather than the drug itself, this high incidence underscores the complexity of treating this vulnerable population. Clinicians must weigh these risks against the potential benefit of avoiding invasive IUTs, particularly given that 42% of neonates also experienced serious complications such as respiratory distress and anemia. Because FcRn also contributes to albumin recycling, albumin‐related safety is clinically relevant. In UNITY, however, no cases of hypoalbuminemia meeting the prespecified adverse‐event threshold (< 20 g/L) were reported. The trial noted only asymptomatic, clinically nonsignificant reductions in albumin, with levels trending back toward baseline after the last dose.

## 3 Discussion

The Phase 2 trial demonstrated the potential of nipocalimab as a transformative treatment for high‐risk pregnancies affected by early‐onset severe HDFN. The primary endpoint—live birth at 32 weeks or later without intrauterine transfusion—was met in more than half of the pregnancies, a result that far exceeded the expectations based on historical outcomes. The reduction in the need for intrauterine transfusions, coupled with delayed transfusions, represents a significant advancement in the management of HDFN.

### 3.1 Comparison With Current Treatments

*Nipocalimab versus Intravenous immunoglobulin (IVIG)* *:* IVIG is commonly used to reduce the need for intrauterine transfusions in HDFN, but its effects are often inconsistent, and many pregnancies still require transfusions [4]. Nipocalimab outperforms IVIG by offering a more potent and consistent mechanism for inhibiting the transfer of alloantibodies.

*Nipocalimab versus Intrauterine Transfusions:* The primary advantage of nipocalimab over intrauterine transfusions is that it reduces or even eliminates the need for transfusions. IUTs are associated with significant risks, mainly when performed before 20 weeks of gestation [5]. By delaying or preventing the need for transfusions, nipocalimab provides a safer alternative for high‐risk pregnancies.

Although nipocalimab represents a significant advance over current standards like IVIG, the next phase of evaluation should be more specific. Beyond confirming efficacy in Phase 3, formal cost‐effectiveness analyses will be important because nipocalimab is a weekly biologic administered over a prolonged period of pregnancy; any economic assessment should weigh drug costs against the potential reduction in repeated IUTs, procedure‐related complications, extreme prematurity, neonatal transfusions, and need of neonatal intensive care unit. Antigen‐specific immunomodulation remains an attractive future strategy because it may suppress only the pathogenic maternal alloimmune response rather than broadly lowering IgG. In that sense, nipocalimab currently appears to be the more clinically mature and near‐term option, whereas antigen‐specific approaches may ultimately offer greater precision if they become technically feasible and affordable. Other antibodies may have some role in treating early HDFN (Table 1). The Phase 3 AZALEA trial is ongoing (Table 2).

**TABLE 1:** Comparative landscape of FcRn inhibitors.

Drug	Type	Mechanism	Placental transfer	HDFN status
Nipocalimab	IgG1 mAb (aglycosylated)	Blocks IgG binding site; negligible effector function.	Limited fetal/neonatal exposure reported in UNITY analyses despite full IgG1 format.	Phase 3 (AZALEA) ongoing; Phase 2 (UNITY) completed. FDA BTD.
Efgartigimod	Fc fragment (engineered)	Mimics Fc‐FcRn interaction; approved for gMG/ITP.	Theoretical transfer profile may differ from full mAbs; human pregnancy/HDFN exposure data remain limited.	Preclinical/early‐phase considerations for HDFN.
Rozanolixizumab	IgG4 mAb	High‐affinity blockade; approved for gMG.	Human placental transfer in HDFN has not been characterized.	Studied in gMG/ITP; potential utility in alloimmune conditions.
Batoclimab	IgG1 mAb	Full mAb blockade.	Placental transfer in pregnancy/HDFN remains unknown.	Clinical trials in gMG, TED, CIDP.

*Note:* Placental‐transfer statements reflect currently available pregnancy/HDFN data and structure‐based expectations; direct head‐to‐head fetal exposure data are lacking for most agents. Nipocalimab’s specific development for HDFN leverages its safety profile in pregnancy, supported by its aglycosylated structure which minimizes immunogenicity and effector risks compared to wild‐type IgG1 formats. Placental transfer may differ across agents according to molecular structure and available pregnancy data.

Abbreviations: BTD, Breakthrough Therapy Designation; CIDP, chronic inflammatory demyelinating polyneuropathy; FcRn, neonatal Fc receptor; FDA, U.S. Food and Drug Administration; gMG, generalized myasthenia gravis; HDFN, hemolytic disease of the fetus and newborn; IgG, immunoglobulin G; ITP, immune thrombocytopenia; mAb, monoclonal antibody; TED, thyroid eye disease.

**TABLE 2:** Comparative analysis: UNITY (Phase 2) versus AZALEA (Phase 3).

Feature	Phase 2 UNITY trial (NCT03842189)	Phase 3 AZALEA trial (NCT05912517)
Study design	Open‐label, single‐arm study. All participants received nipocalimab; no concurrent control group.	Randomized, double‐blind, placebo‐controlled trial. Participants randomized 2:1 to nipocalimab or placebo.
Control/comparator	Historical benchmark. Success is measured against a pre‐specified historical success rate of < 10% for live births without IUT in this high‐risk population.	Standard of care (placebo). The control arm receives standard intensive monitoring and IUTs if anemia develops, enabling direct concurrent efficacy comparison.
Sample size	Small (*N* = 13). Adequate for proof‐of‐concept and safety signal detection, but limited generalizability.	Large (*N* ≈ 120). Powered to provide definitive statistical evidence of efficacy and safety for regulatory approval.
Dosing regimen	Adaptive/exploratory. Evaluated 30 and 45 mg/kg weekly to determine optimal dose.	Fixed high dose. Uses 45 mg/kg weekly dose, confirmed by UNITY to achieve > 95% FcRn receptor occupancy.
Primary endpoint	Live birth ≥ 32 weeks without intrauterine transfusion (IUT). Explicitly focused on avoiding IUT.	Composite event‐free survival. Percentage of pregnancies excluding fetal loss, IUT, hydrops fetalis, or neonatal death—more comprehensive disease control outcome.
Population	Early‐onset severe HDFN. History of IUT or pregnancy loss ≤ 24 weeks.	Severe HDFN risk. Similar inclusion criteria (prior loss or IUT) to ensure high event rates in control arm and adequate power to detect treatment effect.

Abbreviations: FcRn, neonatal Fc receptor; HDFN, hemolytic disease of the fetus and newborn; IUT, intrauterine transfusion; mg/kg, milligrams per kilogram; N, number of participants.

## 4 Conclusion

Nipocalimab represents a promising new treatment for preventing severe early‐onset HDFN in high‐risk pregnancies. Its ability to delay or avoid the need for intrauterine transfusions and its favorable safety profile make it a potentially valuable addition to the management of HDFN. However, further studies with larger sample sizes and more diverse patient populations are needed to confirm its long‐term efficacy and safety.

## Author Contributions

**Aditya Hemedra Bhatt:** writing – original draft, visualization. **Somashekhar Marutirao Nimbalkar:** writing – review and editing, supervision, visualization, validation. **Dipen Vasudev Patel:** writing – review and editing, visualization. **Reshma Kushal Pujara:** writing – review and editing, validation.

## Funding

No specific funding was received for the preparation, authorship, or publication of this Research Highlight. For transparency, the original UNITY clinical trial summarized in this article was funded by Janssen Research & Development. All authors reviewed and approved the final version of the manuscript.

## Conflicts of Interest

The authors declare no conflicts of interest.

## Data Availability

The data that support the findings of this study are available from the corresponding author upon reasonable request.
